# Measuring morbidity and mortality attributable to rheumatic heart disease in Fiji: a prototype study of disease burden in a developing country by data linkage

**DOI:** 10.23889/ijpds.v1i1.385

**Published:** 2017-04-19

**Authors:** Tom Parks, Joseph Kado, Isimeli Tukana, Andrew Steer

**Affiliations:** 1 University of Oxford; 2 Ministry of Health and Medical Services, Fiji; 3 University of Melbourne

## Objectives

Rheumatic heart disease remains a major public health concern in developing countries. Motivated by the lack of up-to-date epidemiologic data from endemic settings, we sought to quantity morbidity and mortality attributable the condition in Fiji, a middle-income country where a high prevalence has consistently been reported. Having resolved to undertake the analysis using the existing routine clinical and administrative data at our disposal, we first set out to develop a data linkage procedure robust to the inherent limitations of data from low resource settings. 

## Approach

Records were available from four sources: an electronic patient information system, a database of death certificates, a disease control register, and echocardiography clinic registers. All referred to 2008-2012.

Throughout the design and calibration process we used 1,406 known duplications in the patient information system from which we calculated the sensitivity and specificity. After cleaning, standardisation and preliminary blocking, we categorised identifiers including names, dates and demographics into agreement, partial agreement, disagreement or missing, accounting for issues such as out of order or misspelt names. After concentrating true matches by further blocking, we estimated match and nonmatch probabilities using expectation maximisation under the Fellegi-Sunter model of record linkage. We then derived the posterior match probability taking into consideration the size of block and prior information about the probability a match be present given the demographics of the individual concerned. In its final configuration, with record pairs considered a match if they achieved a posterior probability of over 50%, our procedure identified the known duplications with sensitivity of 91.4% and specificity of 99.9%. 

## Results

Having identified 2,619 cases from the 1,773,999 records available, we used the linked data to make population-based estimates of prevalence using capture-recapture analyses and cause-specific mortality using relative survival methods, the first such estimates for a developing country. Moreover, in sensitivity analyses, we found that changing posterior probability threshold above which record pairs were considered a match had limited impact on the results. 

## Conclusions

Although data linkage is widely used for epidemiologic research in high-income settings, its application to developing countries has been limited. We developed and validated a data linkage procedure that can be used to turn largely unstudied routine clinical and administrative data into robust estimates of disease burden. With the growing availability of computerized data, we propose our approach has strong potential to assist the production of disease burden statistics in developing countries where civil registration systems are weak.

